# Analytical Device and Prediction Method for Urine Component Concentrations

**DOI:** 10.3390/mi16070789

**Published:** 2025-07-02

**Authors:** Zhe Wang, Jianbang Huang, Qimeng Chen, Yuanhua Yu, Xuan Yu, Yue Zhao, Yan Wang, Chunxiang Shi, Zizhao Zhao, Dachun Tang

**Affiliations:** 1 School of Life Science and Technology, Changchun University of Science and Technology, Changchun 130022, China; wz@cust.edu.cn (Z.W.); 1832175825@mails.cust.edu.cn (J.H.); yu88@mails.cust.edu.cn (Y.Y.); 29500456@mails.cust.edu.cn (X.Y.); 1428124710@mails.cust.edu.cn (Y.W.); 2 School of Optoelectronics Engineering, Changchun University of Science and Technology, Changchun 130022, China; qmchen1989@mails.cust.edu.cn (Q.C.); 2024100339@mails.cust.edu.cn (C.S.); 3 Jilin Institute of Metrology and Science, Changchun 130022, China; 826342489@mails.cust.edu.cn; 4 Changchun Chunqiu Technology Development Co., Ltd, Changchun 130022, China; 444759898@mails.cust.edu.cn

**Keywords:** urine component concentration, whale optimization algorithm, BP neural network

## Abstract

To tackle the low-accuracy problem with analyzing urine component concentrations in real time, a fully automated dipstick analysis device of urine dry chemistry was designed, and a prediction method combining an image acquisition system with a whale optimization algorithm (WOA) for BP neural network optimization was proposed. The image acquisition system, which comprised an ESP32S3 chip and a GC2145 camera, was used to collect the urine test strip images, and then color data were calibrated by image processing and color correction on the upper computer. The correlations between reflected light and concentrations were established following the Kubelka–Munk theory and the Beer–Lambert law. A mathematical model of urine colorimetric value and concentration was constructed based on the least squares method. The WOA algorithm was applied to optimize the weight and threshold of the BP neural network, and substantial data were utilized to train the neural network and perform comparative analysis. The experimental results show that the MAE, RMSE and R^2^ of predicted versus actual urine protein values were, respectively, 3.1415, 4.328 and approximately 1. The WOA-BP neural network model exhibited high precision and accuracy in predicting the urine component concentrations.

## 1. Introduction

Although intelligence technologies have been able to efficiently predict and analyze key parameters in numerous areas, qualitative detection remains the mainstream technique for the dry chemistry dipstick test of urine component concentrations. To be specific, the existing urine detection methods primarily compare the colors of urine test strips with a standard colorimetric card. The principle is to calculate the Euclidean distances from the color values of each test strip to the color blocks with different concentration gradients on the colorimetric card, thereby determining the similarity between the two. When the Euclidean distance is at its minimum, the corresponding colorimetric concentration is identified as the concentration value of the detection item. Although such a method is simple and easy to operate, it still requires improvement in terms of detection range and accuracy [1,2].

In view of this, the present study proposes a fully automated detection device and method for urine test strips, with a view to inferring the specific values of various indices by recognizing the test strip colors. The device obtains the images of reacted reagent blocks via the image acquisition system, extracts the RGB value of each reagent block on the test strip through a series of image processing operations, and then performs color correction and inputs the corrected RGB values into the pre-trained WOA-BP neural network. Through the neural network calculation, the predicted concentration values are obtained, thereby achieving more accurate diagnosis [3,4]. In view of this, the present study proposes a fully automated detection device and method for urine test strips, with a view to inferring the specific values of various indices by recognizing the test strip colors. The device obtains the images of reacted reagent blocks via the image acquisition system, extracts the RGB value of each reagent block on the test strip through a series of image processing operations, and then performs color correction. The corrected RGB values are then input into the pre-trained WOA-BP neural network. Through the neural network’s calculations, the predicted concentration values are obtained, leading to more accurate diagnoses [3,4]. Before the experiment begins, sterile and dry containers should be prepared to avoid the presence of cleaning agents or disinfectants; midstream urine should be collected to minimize contamination from bacteria or impurities at the urethral opening. It is generally recommended to collect morning urine (which is concentrated and stable) or to collect at specific times (such as post-meal urine or random urine). The collected urine should be processed immediately after collection. In respect of centrifugation, if sediment components (such as cells or casts) need to be detected, a sample of 3000 rpm urine should be centrifuged for 5–10 min. If the supernatant needs to be tested, the solution should be well mixed. If the sample is cloudy or has high protein concentration, it should be filtered through filter paper or diluted with physiological saline to avoid interference with the testing equipment or affecting the accuracy of the results. For certain special tests, buffer solutions should be used to adjust the urine pH to an appropriate range to ensure the stability of the test reagents. After completing these steps, the corresponding tests can be conducted.

### Device Design

**Figure 1:**
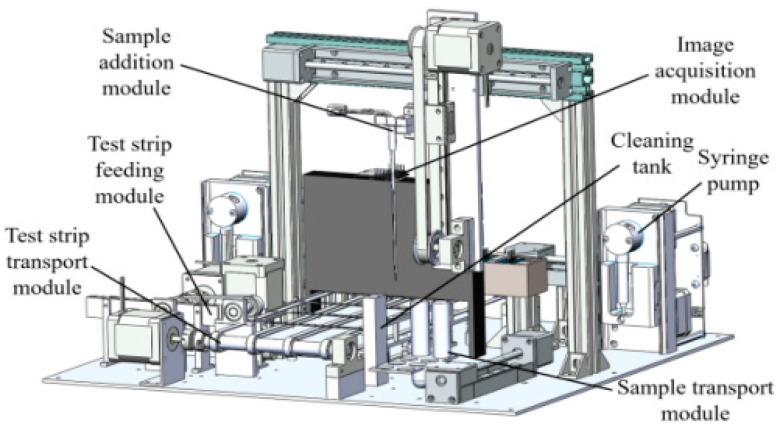
Test strip color recognition device.

Figure 1 illustrates the overall structure of the urine test strip color recognition device, which has a modular design. The functional connection between various parts is realized according to their respective installation and coordination relationships. The device is composed of a test strip feeding module, a test strip transport module, a sample transport module, a sample addition module, a syringe pump, a cleaning tank and an image acquisition module, which can fully automatically complete the test strip placement and transport, sample instillment, image acquisition, image processing and final data analysis.

**Figure 2:**
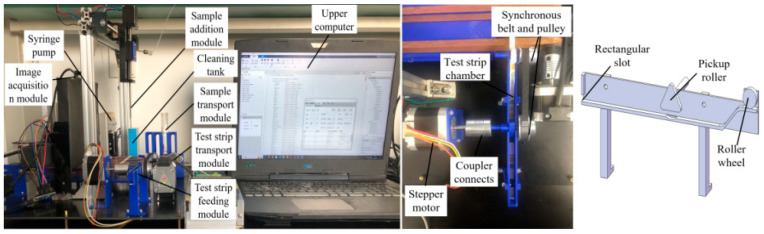
Specific structure of the test strip feeding module.

Figure 2 displays the specific structure of the test strip feeding module, where the test strip chamber, pickup roller, roller wheel and motor support base are all made by 3D printing. The stepper motor provides and controls power for the module; the coupler connects the motor shaft with the pickup roller shaft; and the synchronous belt and pulley transfer power to the roller wheel shaft. The gear ratio of the synchronous belt and pulley is 2:1, so that the pickup roller rotates once and the roller wheel rotates twice. There is a rectangular slot at the rear of the test strip chamber, which facilitates the strip placement. A rubber layer is arranged on the convex part of pickup roller to increase friction. The test strip chamber is provided with a fan-shaped slot under the pickup roller, which enables the pickup roller to press the test strip tightly; the front end is designed with a slope structure to enable the test strip separation. The roller wheel transports a test strip onto the rubber band platform. After the motor shaft rotates counterclockwise to complete the output of single test strip, it needs to rotate clockwise to allow the return of underlying test strip to the origin and then proceeds with the next test strip output.

**Figure 3:**
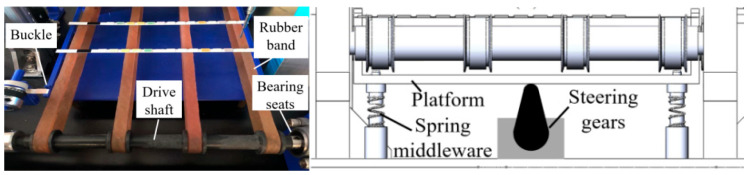
Test strip transport module.

Figure 3 depicts the specific structure of the test strip transport module. The stepper motor provides and controls power for the module; the coupler connects the motor shaft with the drive shafts; and the synchronous belt and pulley transfer power to the roller wheel shaft. The bearing seats offer a fixed support for the drive shaft. The other end of the drive shaft is fixed by two bearing seats, and the fixation of all the bearing seats is achieved by connecting the independently designed 3D printing parts with the bed bolts. The two drive shafts are connected by a rubber band. After the test strip is placed on the rubber band, the two underlying steering gears lift the platform that has a buckle structure. The platform is fixed on the rack by the spring middleware, and the test strip moves on the rubber band platform and gets stuck by the buckle, thereby achieving the strip localization. The steering gear arms lower the platform to allow the passage of the test strip. After the test strip moves for a certain distance, the steering gear arms lift again to ensure a certain inter-strip spacing, which also guarantees that the test strip can stay underneath the sample adding needle and image acquisition module.

**Figure 4:**
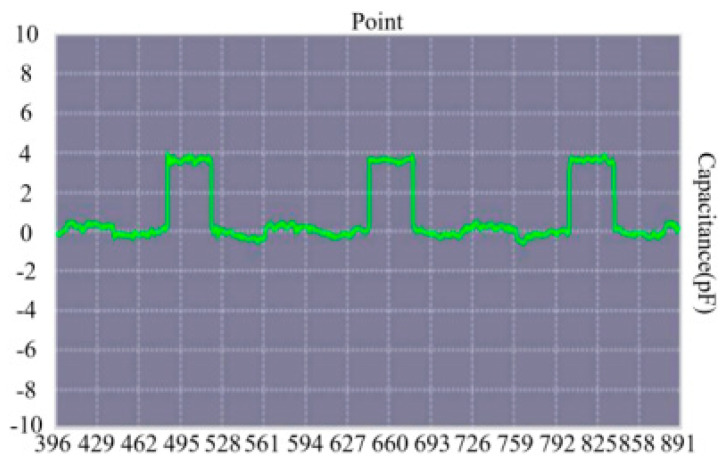
Depicts the waveform of liquid detected under an empty needle condition.

The liquid path system, the core component of the sample addition module, is responsible for ensuring the flow and circulation of urine samples and system liquid during the sample adding process, as well as the disposal of post-reaction waste liquid. Its major functions include multiple important operations such as sample suction and discharge, system liquid delivery and sample-adding-needle cleaning [5]. In the actual sample adding operation, the design and control of the liquid path system is crucial in ensuring the accuracy and efficiency of sample processing. Here, the sample adding needle is required to discharge 20 μL/time repeatedly for 12 times, so its capacity needs to be above 240 μL. Moreover, given the necessity of an air column and a system liquid column, we chose a 400-μL three-layer sample adding needle to effectively isolate the interferences from internal fluid and external space. For liquid level detection, the PCS0902 capacitive level sensor, which has good anti-jamming ability, was selected as the detecting unit. Figure 4 depicts the waveform of the liquid detected under an empty needle condition. MSP1-D1 was adopted as the syringe pump, while a rigid PTFE tube was used for the liquid contact pipeline [6].

## 2. Principle Analyses

### 2.1. Principle of Color Recognition

**Figure 5:**
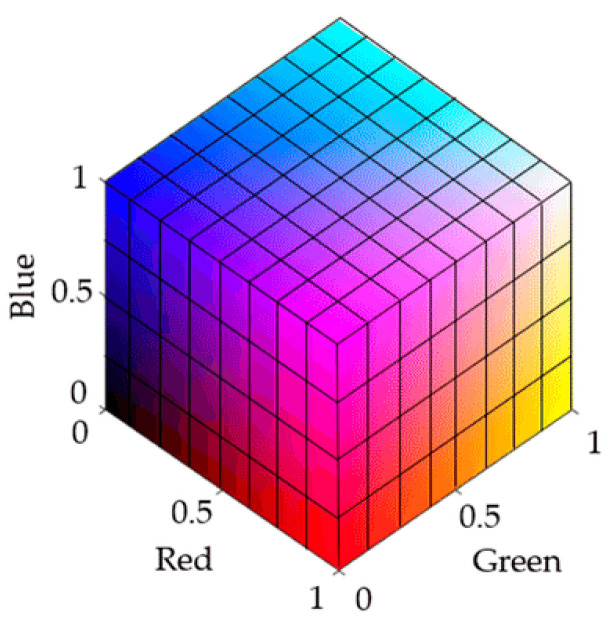
RGB color model.

During the test strip detection, color changes are generated by chemical reactions. Different color space models have been used to recognize and analyze these color changes. Common color spaces include the RGB (red, green, blue) and CIELab spaces. RGB is a device-related color model, and the data acquisition system used in this study was a typical RGB input device. As an addition-based color model, RGB represents various colors by combining the intensity values of three primary colors (R, G, B). The intensity of each primary color is usually expressed as an integer value from 0 to 255. Colors range from completely black (0, 0, 0) to completely white (255, 255, 255), between which different intensities of red, green and blue colors are mixed to form other colors. For the RGB color model displayed in Figure 5, the color range becomes from black (0, 0, 0) to white (1, 1, 1) after normalization [7].

**Figure 6:**
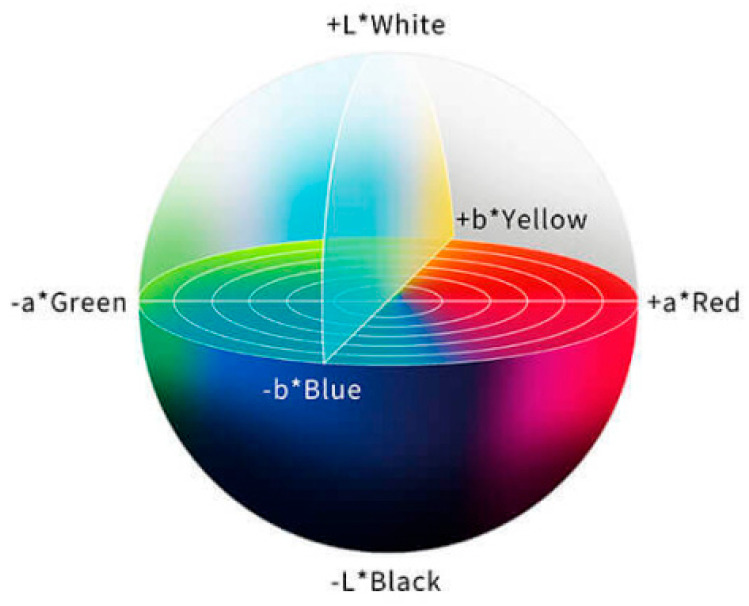
Visual perception-based color space.

The RGB color space may not accurately represent all colors perceived by the human eye, while the CIELab color space is more in line with the understanding of the human visual system, which makes it easier to operate and facilitates color analysis. As a human visual perception-based color space developed by the International Commission on Illumination, CIELab divides colors into three parts, as shown in Figure 6: L* (brightness) stands for the brightness of colors, ranging from 0 (black) to 100 (white); a* (green–red) represents the color distribution from green to red, with negative values indicating green and positive values indicating red; and b* (blue–yellow) represents the color distribution from blue to yellow, with negative values indicating blue and positive values indicating yellow. In the CIELAB color space, the asterisk (*) serves as a specific identifier for the parameters of this color model. CIELab is particularly suitable for detecting small changes in color. For example, when the color of test strip changes from light to dark pink, CIELab can accurately reflect the brightness and tonal difference of such change, thereby facilitating more accurate analysis [8].

The formula for converting a color from RGB to CIELab color spaces is as follows:

Initially, the [R, G, B] value needs to be normalized within the range of [0, 1].

Then, gamma correction is applied to convert this value from nonlinear to linear. For every color channel, the following formula is used:

(1) $$r^{\prime }=\left\{\begin{array}{cc} \frac{r}{12.92} & r\leq 0.04045\\ {\left(\frac{r+0.055}{1.055}\right)}^{2.4} & r>0.04045 \end{array}\right.$$

The same formula applies to $g^{\prime }$ and $b^{\prime }$.

The RGB-to-XYZ conversion matrix is employed as:

(2) $$\left[\begin{array}{c} X\\ Y\\ Z \end{array}\right]=\left[\begin{array}{ccc} 0.4124564 & 0.3575761 & 0.1804375\\ 0.2126729 & 0.7151522 & 0.0721750\\ 0.0193339 & 0.1191920 & 0.9503041 \end{array}\right]\left[\begin{array}{c} r^{\prime }\\ g^{\prime }\\ b^{\prime } \end{array}\right]$$

Subsequently, the XYZ color coordinates are transformed into the CIELab color space.

The normalized X, Y, Z are calculated as:

(3) $$x=\frac{X}{X_{n}},\;y=\frac{Y}{Y_{n}},\;z=\frac{Z}{Z_{n}}$$

where X_n_, Y_n_ and Z_n_ denote the tristimulus values of standard illuminant. In this experiment, X_n_ = 0.95047, Y_n_ = 1.0, and Z_n_ = 1.08883.

*xyz* is transformed into the CIELab color space using the following function *f*(t):

(4) $$f(t)=\left\{\begin{array}{cc} t^{1/3} & t>0.008856\\ \frac{t}{903.3} & t\leq 0.008856 \end{array}\right.$$

The CIELab L*, a* and b* coordinates are calculated as:

(5) $$\begin{array}{l} L^{*}=116\cdot f(y)-16\\ a^{*}=500\cdot (f(x)-f(y))\\ b^{*}=200\cdot (f(y)-f(z)) \end{array}$$

With these formulas, the RGB values can be converted into CIELab coordinates.

### 2.2. Principle of Concentration Calculation

**Figure 7:**
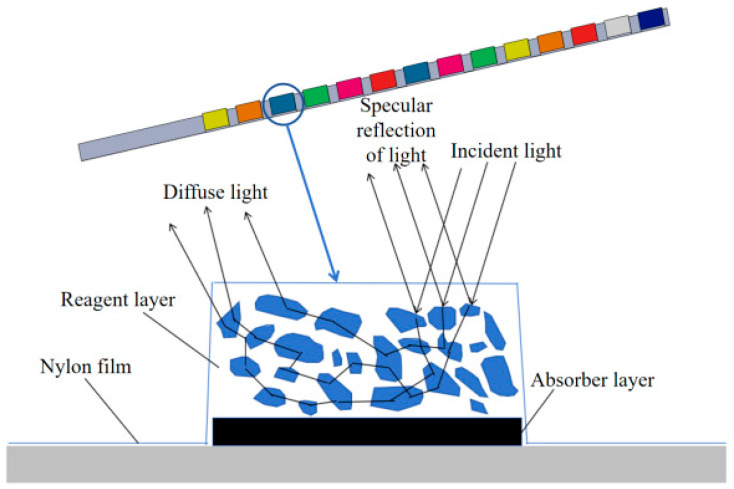
Schematic diagram of reagent block reflection.

Figure 7 illustrates the structure of reagent blocks. The block surface is covered with a nylon film, which can effectively block the macromolecular entry of the reagent layer, thus protecting the reagents from contamination. At the bottom of the reagent blocks, an absorber layer is designed, whose function is to absorb excess urine and prevent incident light from penetrating the reagent layer. Upon contact of the reagent layer with urine, substantial diffuse reflectors would be formed. It can be observed from Figure 7 that specular reflection is produced on the surfaces of the detection blocks, while part of the light enters the diffuse reflectors and eventually forms a diffuse reflection after a series of optical processes such as reflection, refraction and diffraction. When the detection zone of the reagent blocks is sufficiently thick, the influence of transmitted light is negligible. By collecting and analyzing the reflected light, concentration-related detection information can be extracted.

According to the Kubelka–Munk theory, the reflectivity of incident light is specifically correlated with the optical absorption coefficient, the scattering coefficient and the degree of diffuse reflected light absorption in the test strip reaction zone. Such correlation can be formulated as:

(6) $$R=\frac{1}{2}\times [1+\sqrt{1+\frac{4R_{d}}{(1-R_{d}{)}^{2}}}]$$

(7) $$R_{d}=\frac{K}{S}$$

In the above formula, *R* signifies the reflectivity; R_d_ represents the diffuse reflectance when the test sample thickness is greater than the transmission depth; *K* denotes the absorption coefficient of the reagent blocks; and *S* represents the scattering coefficient. Through simultaneous Formulas (6) and (7), we can obtain

(8) $$\frac{K}{S}=\frac{{\left(1-R\right)}^{2}}{2R}$$

The scattering coefficient depends mainly on the object material properties. Thus, when the thickness of the reaction zone and the scattering coefficient remain constant, the reflectivity is only correlated with the absorption coefficient. Since the absorption coefficient *K* and the substance concentration *C* follow the Beer–Lambert law,

(9) K=εC

where *ε* represents the molar absorptivity. The absorption coefficient *K* is linearly proportional to the concentration *C* of the sample being tested. By measuring the reflectance R of the reagent block, quantitative analysis of the target substance concentration in the urine can be achieved.

In the above relation, *ε* denotes the molar absorption coefficient. It is thus clear that the absorption coefficient *K* is directly proportional to the test sample concentration *C*. Hence, as long as the reflectivity of detection reagent blocks is determined, the urine concentrations of corresponding substances can be calculated.

Combining the Kubelka–Munk theory with the Beer–Lambert law, we can derive that reflectivity is directly proportional to concentration. In the ideal state, if a surface is completely diffuse (i.e., the surface reflects all incident light evenly in all directions), the color value of the surface can be regarded as a direct reflection of reflectivity. However, in practical applications, since cameras and sensors are affected by various interfering factors such as lighting conditions and object surface glossiness, the color values cannot directly reflect the reflectivity. Therefore, a direct relationship between color values and concentrations needs to be established through mathematical modeling.

### 2.3. Image Processing

**Figure 8:**
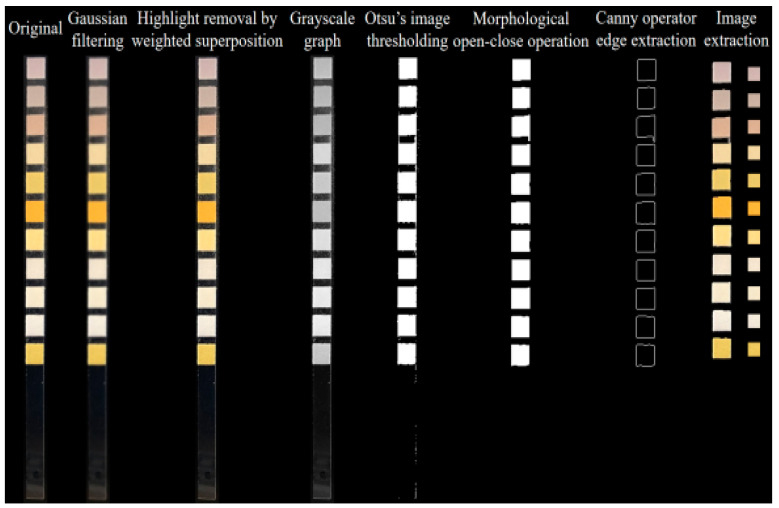
Processing flow of urine test strip image.

A urine test strip image was collected from the image acquisition system, partial functions from the OpenCV library were scheduled in the PyCharm2025.1.1.1 integrated development environment and corresponding program code was written for image processing. Figure 8 schematizes the processing flow, which includes Gaussian filtering, highlight removal by weighted superposition, Otsu’s image thresholding, morphological open-close operation, Canny operator edge extraction, image extraction, color value extraction and color correction. Considering that during the image acquisition, the camera would introduce some noise (predominantly Gaussian) due to device components and various other factors, the Gaussian smoothing filter was used to accomplish image filtering. The highlight removal reduces the influence of highlight zone through linear weighting of the original image with its smooth version (blurred image). During threshold segmentation, the Otsu’s method was employed to automatically obtain the image thresholds, and the optimal threshold was calculated automatically based on the image gray distribution, thereby separating the background region from the foreground region. The core idea of Otsu’s method is to maximize the variance between classes and find a gray-level threshold T that maximizes the separation between foreground and background pixels. The first step involves calculating the histogram and probability distribution. Assuming the gray level range is from 0 to L − 1, the probability of the i-th gray level is:

(10) $$P_{i}=\frac{\mathrm{The}\;\mathrm{number}\;\mathrm{of}\;\mathrm{pixels}\;\mathrm{with}\;\mathrm{pixel}\;\mathrm{value}\;i}{\mathrm{Total}\;\mathrm{number}\;\mathrm{of}\;\mathrm{pixels}}$$

For a given threshold T, the Otsu method divides the image into two categories: background pixels (gray levels [0, T]), with probability $\omega_{0}$ and average gray level $\mu_{0}$; and foreground pixels (gray levels [T, L − 1]), with probability w1 and average gray level $\mu_{1}$. The inter-class variance is defined as:

(11) $$\sigma_{b}^{2}=\omega_{0}\omega_{1}{\left(\mu_{0}-\mu_{1}\right)}^{2}$$

(12) $$\begin{array}{l} \omega_{0}=\sum_{i=0}^{T-1}P(i)\\ \omega_{1}=\sum_{i=T}^{L-1}P(i)\\ \mu_{0}=\frac{\sum_{i=0}^{T-1}i\cdot P(i)}{\omega_{0}}\\ \mu_{1}=\frac{\sum_{i=T}^{L-1}i\cdot P(i)}{\omega_{1}} \end{array}$$

By traversing all possible threshold values of T, Ot finds the T that maximizes σb2. This T value is the optimal segmentation threshold.

For morphological processing, the opening operation was performed on the image first to eliminate some small-pixel interfering color blocks. Then, closing operation was performed to fill the small holes in the target color block zones. The primary purpose of morphological processing is to segment the independent elements of the image and reduce the interferences in small and medium-sized regions therein. After the above image operations, the edges of each urine dry chemistry test strip image were sharp and easy to locate. Thus, the Canny operator was directly applied to extract the image edges, obtaining the edge positions of various reagent blocks on the test strip. The image center was determined based on the edges, and by extracting rectangular images with a certain pixel size from the central position, we could obtain respective images of each detection item. Finally, the average RGB value of pixels in the region was calculated, thereby acquiring the representative color information of each reagent block.

**Figure 9:**
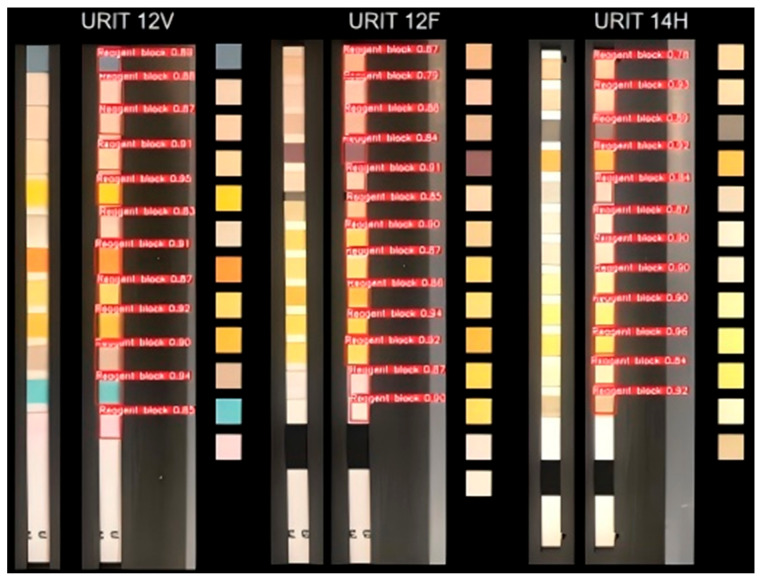
the detection effect of the trained model.

In addition to the conventional image processing methods, the YOLOv5 model was also used to train and detect the test strip images. As an advanced object detection model, YOLOv5 has been widely applied in image recognition and localization tasks, and is capable of quickly and accurately identifying objects and their positions in images. Substantial urine test strip images were collected and each detection item color block in the images was accurately annotated with Makesense. These annotated data were utilized to train the YOLOv5 model, allowing it to accurately identify the color block zones of all detection items in the test strip image. Figure 9 describes the detection effect of the trained model. Regardless of the type of test strip, the model exhibits good detection performance, which can accurately identify the location of each reagent block. During the detection process, the model assigns a confidence value to each detected target zone, which is used for measuring the reliability of detection results. In this study, a detection region with a confidence level of 0.78 or above is regarded as an effective target region and is labelled a “reagent block”. Further processing was carried out on these target regions. Initially, the coordinates of the center point of the detection box were extracted. Then, an image area with a pixel size of 15 × 15 was extracted centering on this central point, which served as the pure color block image of corresponding detection items [9,10].

**Figure 10:**
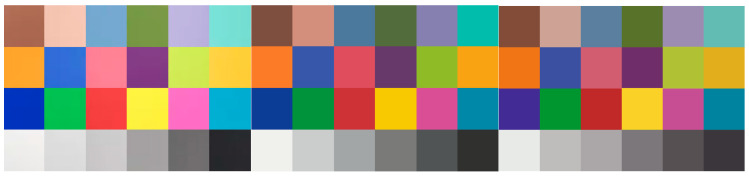
Color block images collected by the device.

Given the characteristics of the image acquisition system and the influence of environmental factors, the directly extracted color values may have certain deviations. Thus, a final color correction process is required. Through color correction, the extracted color values can be adjusted and corrected, thereby obtaining more accurate color values. The color correction here is specifically the device color correction. By fitting the mapping relationship between the color values captured by the image acquisition system and the known color values on a standard colorimetric card, a polynomial regression model was constructed to correct the color deviations from the device. Initially, it is necessary to photograph the international standard colorimetric card in a fixed lighting environment. All the obtained data were converted from the RGB color space to the CIELab color space with Formulas (11) and (12). The Lab values of the color blocks collected by the system, the known Lab values of standard color blocks, and the corresponding color block images before and after polynomial nonlinear correction are presented in Figure 10. For every color channel (L, a, b), a polynomial fitting model was built as:

(13) $$x_{s}=a_{0}+a_{1}x_{m}+a_{2}x_{m}^{2}+\ldots +a_{n}x_{m}^{n}$$

where *x_s_* signifies the standard value of color channel; *a*_0_, *a*_1_, …, *a_n_* represent the fitted polynomial coefficient; *x_m_* denotes the measured value of color channel; and *n* is the polynomial order. Given the standard colorimetric card dataset, the polynomial coefficients *a_i_* of various channels were fitted by the least squares method as follows:

(14) $$\underset{a_{0},a_{1},\ldots ,a_{n}}{\min}\sum_{i=1}^{m}(x_{s}-(a_{0}+a_{1}x_{i}+a_{2}x_{i}^{2}+\ldots +a_{n}x_{i}^{n}){)}^{2}$$

where *x_i_* represents the device measured value and *m* denotes the sample number of standard colorimetric card.

The specific implementation process in the program code is as follows: a NumPy library was used for array operation. The focus was on constructing polynomial features by scheduling the preprocessing function in a sklearn library, thereby extending the input data to polynomial features. For example, when degree = 2, the input data x would be extended to [1, x, x^2^]. The linear_model function in the sklearn library was applied to fit the polynomial regression equation, and the regression model was trained using the measured extended polynomial feature matrix X_poly and reference target values, thereby obtaining the weight coefficient and intercept of each polynomial feature. The polyfit function code for single-channel data is as follows:

//Polynomial fitting of single-channel data

# Scheduling corresponding function library

from numpy.polynomial.polynomial import Polynomial

from sklearn.preprocessing import PolynomialFeatures

from sklearn.linear_model import LinearRegression

import numpy as np

# Single-channel fitting function

def fit_polynomial(measured, reference, degree = 2):

# Creating polynomial features

poly = PolynomialFeatures(degree)

X_poly = poly.fit_transform(measured.reshape(−1, 1))

X_poly = poly.fit_transform(measured.reshape(−1, 1)) # Constructing polynomial features

# Fitting polynomial regression model

model = LinearRegression().fit(X_poly, reference)

return model

It is necessary to separately calculate the corresponding polynomial models for the three color channels L, a and b. Substituting the Lab values of the color block images into the model yields corrected Lab color values that are close to the standard. Figure 10, from left to right, shows the schematic diagram of device color block acquisition, the original image of the standard color chart and the effect diagram of polynomial nonlinear correction.

G_1_B_1_, R_2_G_2_B_2_, R_3_G_3_B_3_, …, R_i_G_i_B_i_ values corresponding to various concentration levels (−, −+, +, ++, +++, ++++) of corresponding items on the standard colorimetric card of urine test strip were separately calculated. The computational formula for the Euclidean distance ΔE_ab_ is:

(15) $$\Delta E_{ab}=\sqrt{{\left(L^{*}-L_{i}^{*}\right)}^{2}+{\left(a^{*}-a_{i}^{*}\right)}^{2}+{\left(b^{*}-b_{i}^{*}\right)}^{2}}$$

Through comparative calculation, all the CIELab distance values ΔE_ab_ were obtained. The concentration level on the standard colorimetric card corresponding to the smallest ΔE_ab_ was precisely the concentration level of the urine test strip detection item.

### 2.4. Regression Analysis

**Figure 11:**
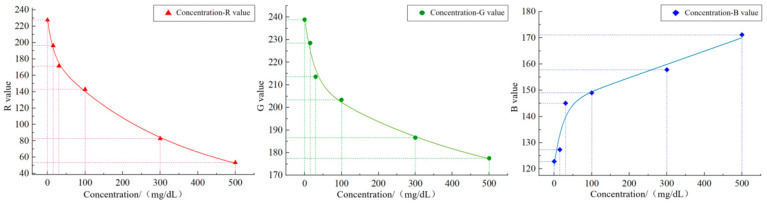
Protein concentration–RGB value fitting relationship.

**Figure 12:**
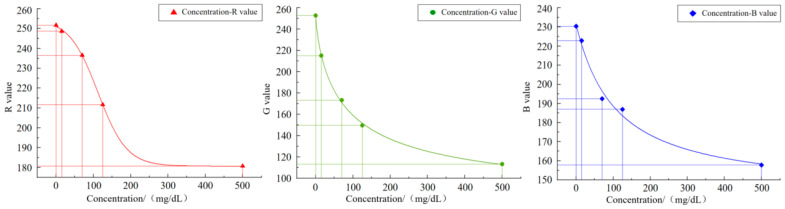
Leukocyte concentration–RGB value fitting relationship.

The image acquisition system collects *RGB* color values from the protein and leukocyte items of the urine test strip colorimetric card and uses them as the input features after color correction. In OriginPro2018, the nonlinear fitting relationships between the *R*, *G* and *B* values corresponding to each collected protein concentration and the concentration value were separately constructed, as described in Figure 11 and Figure 12. The fitting equations of the two are presented in Table 1 and Table 2, where *C* denotes the concentration value. The *R^2^* values of the three equations are all above 0.95, proving a good fitting effect. Since this model roughly describes the variation trend of concentration value with the color values, a more accurate mathematical model is required to predict the corresponding concentration value based on the three color values.

For the sample acquisition, a concentration value was input into the above three regression equations to obtain a set of data comprising an RGB value and a concentration value C. After multiple calculations, the obtained data were divided into the training set (500 data) and the test set (70 data).

### 2.5. Whale Optimization Algorithm (WOA)

WOA, first proposed by Seyedali Mirjalili et al. in 2016, is an optimization algorithm that simulates whale behavior. The core idea stems from humpback whales’ unique bubble-net feeding strategy. When humpback whales hunt, they blow spiraling circles of bubbles to create a net around their prey, gathering the prey up for easy capture. By simulating this process, WOA searches for the optimal solution in the solution space. The location of each whale corresponds to a potential solution, and the global optimal solution is gradually approached by constantly updating the whale locations. This predation process consists of three stages: the prey encirclement, the bubble-net assaulting and the prey search.

The behavior of encircling prey is modeled by calculating the distance between the whale and the prey (current optimal location) and adjusting the whale location according to this distance [11,12,13]. The specific location update formula is:

The distance vector *D* between the current whale location and the optimal location is determined as:

(16) $$D=\left|C\cdot X^{*}(t)-X(t)\right|$$

(17) $$C=2\cdot r_{2}$$

where *C* stands for a coefficient vector that adjusts the search range, with *r*^2^ being a random number between [0, 1]. *t* denotes the number of iterations; X^*^(*t*) represents the current global optimal location; and *X*(*t*) represents the current whale location.

The adjustment vector *A* is calculated to determine the offset of the whale location relative to the optimal location:

(18) A=2a⋅r−a

(19) $$a=2-t\cdot \frac{t}{T_{\max}}$$

*a* is a coefficient that decreases linearly from 2 to 0 to control the search convergence process; *r* is a random number between [0, 1]; and *T_max_* denotes the maximum number of iterations.

Using the distance vector *D* and the adjustment vector *A*, the whale location *X*(*t* + 1) is updated as:

(20) $$X(t+1)=X^{*}(t)-A\cdot D$$

During the bubble-net assaulting behavior, the whale updates its location by calculating its distance from the optimal location and by gradually approaching the optimal location along the spiral path. The specific location update formula is:

The distance vector *D* between the current whale location and the optimal location is calculated as follows:

(21) $$D^{\prime }=\left|X^{*}(t)-X(t)\right|$$

Using the distance vector *D* and a spiral path, the whale location *X*(*t* + 1) is updated as follows:

(22) $$X(t+1)=D^{\prime }\cdot e^{bl}\cdot \cos(2\pi l)+X^{*}(t)$$

where *e^bl^* represents an *l*-dependent exponential function that simulates the bubble-net *c*ontraction or expansion; cos(2π*l*) generates an *l*-dependent cosine function to simulate the spiral motion; *b* is a constant that determines the spiral shape; and *l* is a random number between [−1, 1].

When |*A*| < 1, the probability parameter *p* is compared with the preset threshold to identify which of the above behaviors a whale specifically chooses for location updating.

*p* is a random number between [0, 1]. If *p* < 0.5, the whale chooses to encircle the prey, which is suitable for the initial stage when a large-area search is required. If *p* ≥ 0.5, bubble-net assaulting behavior is chosen, which is more suitable for the later stage of the algorithm and enables more accurate approximation when approaching the optimal solution.

When |*A*| ≥ 1, under the prey search behavior, the specific location update formula of whale is as follows:

(23) $$X(t+1)=\left\{\begin{array}{cc} X^{*}(t)-A\cdot D, & if\;p<0.5\\ D^{\prime }\cdot e^{bl}\cos(2\pi l)+X^{*}(t), & if\;p\geq 0.5 \end{array}\right.$$

where *X^rand^*^(*t*)^ is the location of a whale randomly selected from the whale population.

## 3. Experimental Results and Discussions

### 3.1. Network Construction

**Figure 13:**
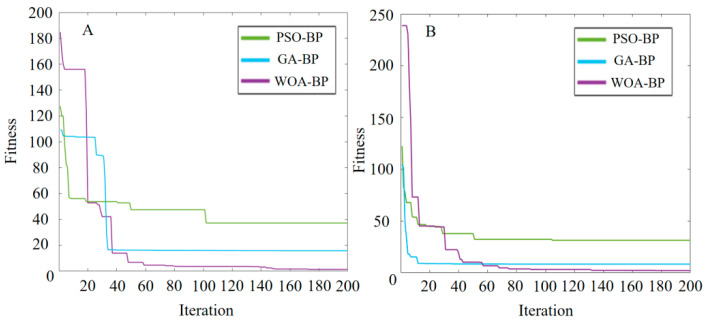
Fitness iteration curves of networks optimized by different algorithms. (**A**) Proteins. (**B**) Leukocytes.

The specific process of optimizing a BP neural network using the WOA is described in Figure 13. The relevant steps are as follows: (1) data were subjected to normalization pre-processing; (2) optimal network topology was determined by exhaustive method; (3) the BP neural network was initialized, including determination of the network input and output structures, initial connection weights and thresholds; (4) the WOA population was randomly initialized, where each individual represented the weight and threshold of a set of BP neural networks; (5) WOA calculation was performed by taking the training error of the BP neural network as the fitness value and the network weight and threshold as the population individual; (6) the above optimization and updating process was repeated to gradually approach the optimal population location and the minimum fitness value (iterative updating); (7) the optimal network weight and threshold were obtained through the optimization process; (8) the network was trained using the optimized network weight and threshold; and (9) after completion of the training, the optimized neural network was used to output the prediction results.

### 3.2. Network Training

The WOA-BP neural network was trained according to the above parameter settings. Meanwhile, the particle swarm optimization (PSO) algorithm and the genetic algorithm (GA) were employed to optimize the BP neural network, and the training effect was compared with that of the WOA-BP neural network [14,15]. The fitness iteration curves of protein and leukocyte items are presented in Figure 13. Clearly, the three optimization algorithms can all significantly enhance the searching fitness and improve the model performance, especially the WOA-BP algorithm, which exhibits the fastest convergence and the lowest final fitness during the iterative process. According to Table 3, the MAEs and RMSEs of BP neural networks optimized by the three algorithms all decreased significantly on the test set. Compared with the PSO and GA, the WOA outperformances in terms of MAE, RMSE and fitness, suggesting that it has the fastest convergence during optimization, the highest prediction accuracy and the best prediction effect.

### 3.3. Results Analysis

**Figure 14:**
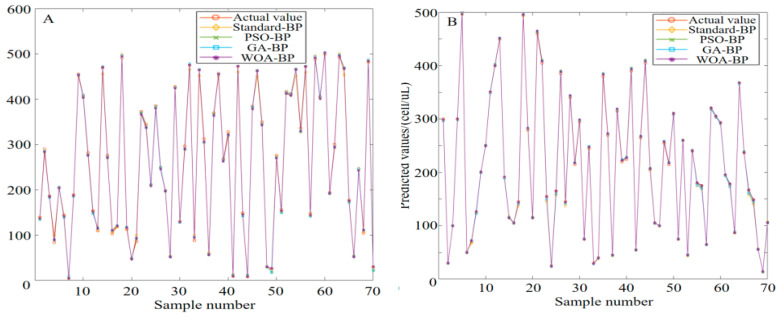
Predicted value comparison among networks optimized by different algorithms. (**A**) Proteins. (**B**) Leukocytes.

**Figure 15:**
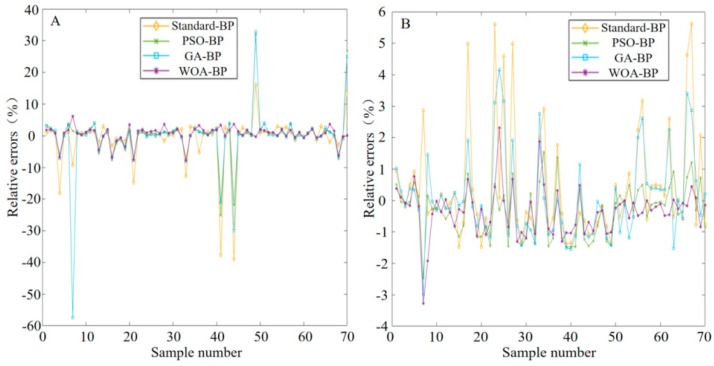
Prediction error comparison among networks optimized by different algorithms. (**A**) Proteins. (**B**) Leukocytes.

The prediction results of protein and leukocyte concentrations in urine using various algorithms are presented in Figure 14. Clearly, the predicted values of the four optimization algorithms are very close to the actual values. In particular, for three algorithms—PSO-BP, GA-BP and WOA-BP—their predicted values almost coincide with the actual values, indicating their excellence in prediction accuracy. Contrastively, although the predicted value of the standard BP algorithm is also close to the actual value, some deviations exist on some samples. Since these two devices are conducting simultaneous and parallel detection, the total response time should be taken as the maximum value between the two, which is 52.0 ± 2.5 s, and it is less than 60 s. Figure 15 displays the prediction errors of various algorithms. Based on analysis combining the two figures, the prediction errors of PSO-BP are small and stable, with relative errors ranging mostly between −10% and 10%. GA-BP also exhibits small prediction errors on most samples, but larger errors of nearly −60% on individual samples (e.g., protein number 7). The overall prediction error of WOA-BP is minimal and stable, with relative errors mostly concentrated between −5% and 5%.

The predicted protein results were verified and compared with the manual detection results. For the preparation of a 500 mg/dL bovine serum albumin (BSA) solution, 0.5 g of BSA powder was weighed first with a precision electronic balance and then placed into a sterile 50 mL centrifuge tube. After slowly adding 30 mL of sterile 1×PBS buffer, the centrifuge tube was shaken slightly to allow preliminary dissolution of the powder. Subsequently, the centrifuge tube was turned upside down slowly to mix the solution uniformly until the powder was fully dissolved. No bubbles should be generated during the whole process. The fully dissolved solution was transferred into a 100 mL volumetric flask, and then sterile 1×PBS buffer was added to the mark. Finally, the volumetric flask was turned upside down slowly several times to ensure that the solution was mixed uniformly [16]. The prepared solution was stored in a 4 °C refrigerator. The preparation method of protein solutions for the remaining concentration gradients was the same as above. The urine protein detection was based on the principle of “protein error of pH indicators”. The reagent blocks used for protein detection contained the tetrabromophenol blue indicator and the buffer. The positive charge energy of proteins could bind to the tetrabromophenol blue anions, causing the pH alteration to produce a color change from yellowish green to bluish green. At low concentrations, the test strip was yellow or light green, and as the concentration rose, the color gradually deepened to bluish green. For each gradient, 20 μL was instilled onto the reagent blocks. After reacting for 30 secs, the reagent blocks were placed in the data acquisition system to read the corresponding RGB values. The data for each gradient were averaged from ten acquisitions. These RGB values were corrected and imported into the neural network as input features. Through prediction, the corresponding concentration results were obtained. Table 4 details the prediction results of test samples.

**Figure 16:**
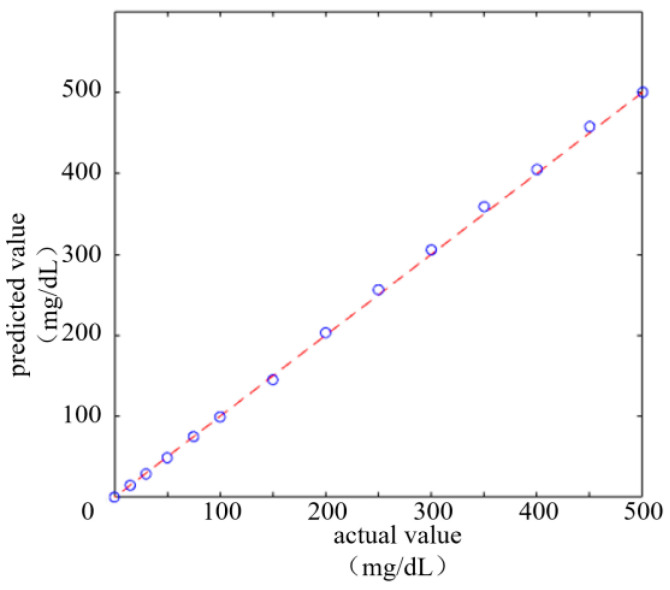
Goodness of fit curves.

Figure 16 displays the goodness of fit results drawn based on data in the above table, where the dashed diagonal line represents the consistency between actual and predicted values under ideal conditions, while the scatter dots represent the ratio of each actual value to the corresponding predicted value. These scatter distribution trends are very close to the diagonal line, indicating a good consistent regression between the model predicted results and the actual values. Meanwhile, the measured results were computationally analyzed. The MAE, RMSE and *R*^2^ of the predicted versus actual values were 3.1415, 4.328 and 0.99931, respectively. The *R*^2^ value was superior to that of the regression of concentration against RGB values, suggesting that the average deviation between the model predicted results and actual values was small, and that the model had a strong ability to interpret the real data.

## 4. Conclusions

In this study, a fully automated detection device for urine test strips was successfully designed, and a urine component concentration analysis system based on the image acquisition module and WOA-BP neural network was constructed. The device automatically drops samples onto the test strip, the image acquisition module collects the color information of the test strip, and the WOA-BP neural network performs quantitative regression prediction, thus achieving accurate prediction of the urinary protein and leukocyte concentrations. The experimental results show that the analysis system has high accuracy and reliability in predicting the concentration of urine samples, with small MAE and RMSE between the predicted and actual values, and the R^2^ coefficient approximately 1. These indicate that the model has a strong predictive ability. The proposed detection method only analyzes the protein and leukocyte items on the test strips. Different detection items of different test strips need to be analyzed differently, where training of different networks is required, resulting in a huge time cost. The present study provides a new idea and method for the development of urine dipstick detection, which has certain theoretical and application value. In the future, the hardware performance and optimization algorithm can be further improved, and the sample data and diversity of test strip detection items should be further expanded to enhance the model applicability, with a view to meeting broader clinical needs.

## Figures and Tables

**Table 1:** Regression equation of protein concentration–RGB value fitting relationship.

Category	Regression Equation	R^2^
C-R	*y* = 45.17 × exp(−*x*/15.67) + 170.50 × exp(−*x*/345.99) + 12.55	0.9983
C-G	*y* = 27.86 × exp(−*x*/24.47) + 53.51 × exp(−*x*/484.70) + 158.29	0.9919
C-B	*y* = −23.29 × exp(−*x*/23.61) – 707,813.13 × exp(−*x*/1.39) + 707,957.78	0.9578

**Table 2:** Regression equation of leukocyte concentration–RGB value fitting relationship.

Category	Regression Equation	R^2^
C-R	*y* = 180.69 + 74.17/(1 + exp((x − 112.27)/38.33))	0.9900
C-G	*y* = 77.88 + 174.77/(1 + (x/83.35)^0.77)	0.9991
C-B	*y* = 143.66 + 87.49/(1 + (x/105.65)^1.02)	0.9904

**Table 3:** Parameter comparison among networks optimized by different algorithms.

Protein	Mae	Rmse	Fitness	Leukocyte	Mae	Rmse	Fitness
Standard BP	4.4254	6.0815	/	Standard BP	2.1294	3.0597	/
PSO-BP	3.4683	4.2069	37.2043	PSO-BP	1.0384	2.4665	31.3348
GA-BP	3.3687	4.1220	15.7919	GA-BP	0.7124	2.311	8.3059
WOA-BP	3.1503	3.8618	1.3161	WOA-BP	0.1836	1.8119	1.535

**Table 4:** Prediction results of test samples.

Serial Number	True Concentration Value mg/dL	Input the RGB Values of the Network			Predicted Concentration Value mg/dL
		R	G	B	
1	500	53.35	177.52	171.15	500.0475
2	450	58.99	179.44	167.47	457.5366
3	400	66.21	181.74	164.93	404.6016
4	350	74.55	184.28	162.4	358.8658
5	300	82.64	186.66	157.8	305.5325
6	250	95.33	190.24	157.33	256.276
7	200	108.2	193.72	154.78	203.2454
8	150	123.08	197.62	152.21	145.2632
9	100	142.94	203.39	149.06	99.3458
10	75	150.2	205.43	147.48	75.0586
11	50	161.97	210.17	144.37	48.9074
12	30	171.31	213.58	145.07	29.0233
13	15	196.36	228.5	127.35	15.0114
14	0	227.63	238.84	122.85	0.3451

## Data Availability

The datasets presented in this article are not readily available because the data are part of an ongoing study and due to technical limitation]. Requests to access the datasets should be directed to 2024101566@mails.cust.edu.cn.
